# Prognostic importance of septal deformation patterns and lateral wall function in patients with heart failure and left bundle branch block receiving cardiac resynchronization therapy

**DOI:** 10.1093/ehjimp/qyag066

**Published:** 2026-05-13

**Authors:** Niels Risum, Emil Anton Frandsen, Bhupendar Tayal, Thomas Fritz Hansen, Michael Vinther, Berit Thornvig Philbert, Trine Kiilerich Lauridsen, Ulrik Winsløw, Samir Saba, Joseph Kisslo, John Gorcsan, Peter Sogaard

**Affiliations:** Department of Cardiology, The Heart Centre, Copenhagen University Hospital—Rigshospitalet, 2142, Inge Lehmanns Vej; Department of Cardiology, The Heart Centre, Copenhagen University Hospital—Rigshospitalet, 2142, Inge Lehmanns Vej; Division of Cardiology, University of Arkansas for Medical Sciences, Little Rock, AR, USA; Department of Cardiology, Copenhagen University Hospital—Herlev and Gentofte, Herlev, Denmark; Department of Cardiology, The Heart Centre, Copenhagen University Hospital—Rigshospitalet, 2142, Inge Lehmanns Vej; Department of Cardiology, The Heart Centre, Copenhagen University Hospital—Rigshospitalet, 2142, Inge Lehmanns Vej; Department of Cardiology, Copenhagen University Hospital—Herlev and Gentofte, Herlev, Denmark; Department of Cardiology, The Heart Centre, Copenhagen University Hospital—Rigshospitalet, 2142, Inge Lehmanns Vej; Division of Cardiology, University of Pittsburgh Medical Center, Pittsburgh, PA, USA; Division of Cardiovascular Medicine, Duke University Medical Center, Durham, NC, USA; Heart and Vascular Institute, Penn State University College of Medicine, Hershey, PA, USA; Department of Cardiology, Aalborg University Hospital, Aalborg, Denmark

**Keywords:** longitudinal strain analysis, long-term outcome, cardiac resynchronization therapy, left bundle branch block, septal deformation patterns

## Abstract

**Aims:**

A typical left bundle branch block (LBBB) contraction pattern prior to cardiac resynchronization therapy (CRT) has been demonstrated to be highly associated with response beyond QRS morphology and duration. This study investigates whether septal deformation type and lateral wall strain amplitude may be of particular importance for long-term outcome after CRT implantation.

**Methods:**

From two centres, 208 CRT candidates with LBBB, New York Heart Association functional class II–IV, left ventricular ejection fraction ≤35%, and QRS duration ≥ 120 ms underwent echocardiography before CRT-defibrillator (CRT-D) implantation.

**Results:**

Four septal contraction patterns were identified: 36 patients (17%) had double-peaked systolic pattern (LBBB-1), 51 (25%) had early pre-ejection peak shortening followed by systolic stretch (LBBB-2), 43 (21%) had shortening with one systolic peak inside 70% of the ejection phase (LBBB-3), and 78 (38%) had normal septal peak timing outside early 70% of ejection phase (LBBB-4). The primary outcome (freedom from death, implantation of a left ventricular assist device, and heart transplantation) occurred in 48 patients during a median follow-up of 4 years (interquartile range 3.25–4). The most favourable outcome was associated with LBBB-1 and LBBB-2, which had one-seventh the risk of the primary outcome compared to LBBB-4 (HR: 0.14 [95% confidence interval: 0.06–0.34]; *P* < 0.001). Patients in the lowest tertile of lateral wall strain (>−7.4%) were at high risk of an event compared to the other patients (HR: 2.57 [1.46–4.55]; *P* = 0.001).

**Conclusion:**

The long-term clinical benefit of CRT-D in patients with LBBB pre-implantation varies. Subtypes of septal contraction patterns and, to a lesser degree, lateral function are important determinants of outcome in patients with CRT-D.

## Graphical Abstract

**Graphical Abstract:**
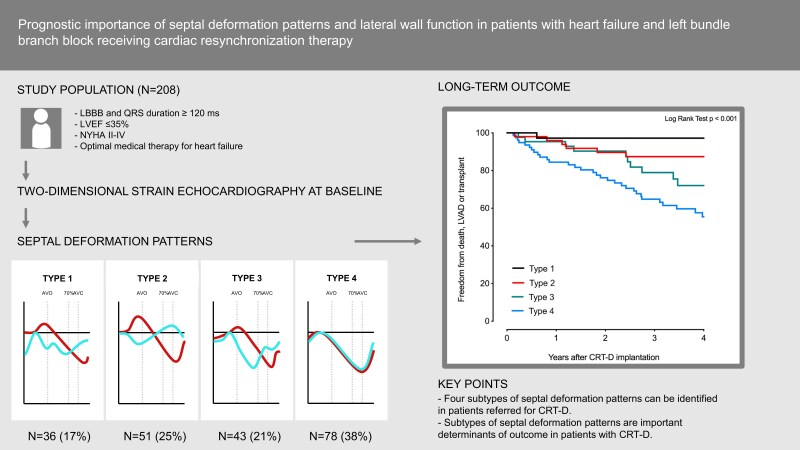

## Introduction

Successful cardiac resynchronization therapy (CRT) favours patients with left bundle branch block (LBBB) by electrocardiography (ECG).^1^ However, not all LBBB´s by ECG are the same.^2,3^ Studies show that one-third of patients with LBBB by ECG do not have a significant activation delay in the left ventricle (LV),^4,5^ and patients with LBBB by ECG show highly variable responses after CRT.^6^ In order to identify true LBBB activation, we have proposed the use of two-dimensional (2D) strain echocardiography to specifically detect the characteristic opposing wall contraction associated with LBBB activation.^3,7^ LV wall motion involves early peak contraction of the septal wall, early stretching of the lateral wall, and delayed lateral wall peak contraction.^8,9^ This LBBB contraction pattern prior to CRT is highly associated with favourable LV remodelling and long-term survival beyond ECG morphology and duration.^10^

Although a typical strain pattern described in our previous work is simple, in practice, different configurations can be identified that may be important for long-term outcome. Computer simulations have demonstrated how septal deformation patterns derive integrated information of both electromechanical dyssynchrony and regional LV contractility, both of which are important determinants of a favourable response to CRT.^11^ Studies have indicated a role for the septal deformation patterns in the prediction of LV reverse remodelling and mortality.^12–14^ However, a longer follow-up period is needed to evaluate the predictive value of these patterns on long-term CRT outcomes.

In an attempt to further understand the prognostic significance of LBBB contraction throughout systole, the predictive ability of subtypes of septal deformation patterns and the lateral wall performance was investigated by 2D strain echocardiography with 4 years of follow-up.

We hypothesized that

1. Subtypes of septal deformation patterns are independently associated with outcome.
2. Lateral wall deformation amplitude is independently associated with outcome.
3. Regional septal and lateral strain markers are superior to global LV function in the prediction of outcomes.

## Methods

Overall, 234 consecutive patients with LBBB were prospectively included from 2010 to 2011 (139 patients from the University of Pittsburgh Medical Center and 95 patients from Gentofte University Hospital, Denmark) as previously described.^10^ All patients had LBBB by conventional ECG criteria^15^ and fulfilled the following criteria: left ventricular ejection fraction (LVEF) ≤ 35%, QRS duration ≥120 ms, and New York Heart Association functional class II–IV despite optimal pharmacologic therapy for heart failure. Informed consent was obtained from all participants in the study.

All patients had a three-view segmental strain dyssynchrony study performed prior to CRT for research purposes. The study was not used for decision-making with regard to CRT implantation in any of the patients.

Clinical data, baseline data, and laboratory work were collected for all patients. Exclusion criteria were significant primary valve disease, right ventricular pacing, atrial fibrillation, or acute coronary syndrome or revascularization within 3 months of the baseline echocardiography.

Patients were implanted with a CRT device with defibrillator capacity (CRT-D) according to the standard clinical practice. The atrial lead was placed in the high right atrium, and patients received a right ventricular apical or septal lead, and an LV-lead positioned through the coronary sinus in an epicardial vein targeting posterolateral or lateral branches when possible. The study protocol was approved by the institutional review board at both centres and complied with the Declaration of Helsinki.

### Echocardiography

Prior to CRT a full standard echocardiographic examination, including grayscale images optimized for 2D strain analysis (mean frame rate 62 ± 17 frames/sec) was performed. All echo studies were acquired with a Vivid 7 Dimension or Vivid 9 ultrasound machine using a 3.5 MHz ultrasound probe (General Electric (GE)-Vingmed Ultrasound, Horten, Norway.) Off-line analyses were performed using EchoPac PC version BT11 (GE-Vingmed Ultrasound) by two experienced readers. The LV end-systolic volumes (ESV), end-diastolic volumes (EDV), and LVEF were assessed using the biplane Simpson method.

### Two-dimensional strain echocardiography

**Figure 1:**
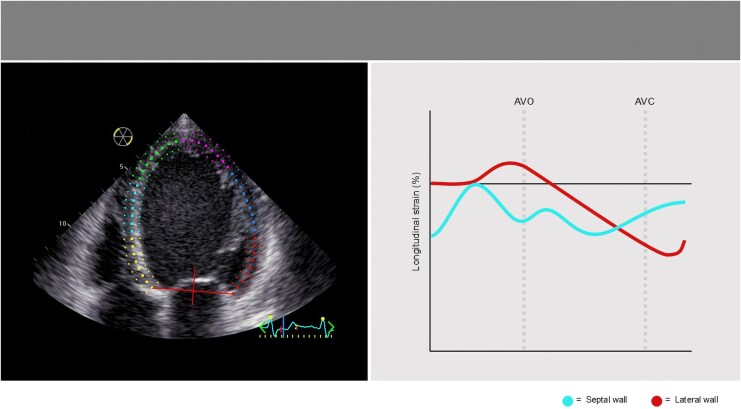
Measurement of longitudinal strain. The right panel shows strain traces from the septal (cyan) and lateral (red) wall. AVO; aortic valve opening. AVC; aortic valve closure.

A 2D strain analysis was performed in the three apical views. The most suitable cardiac cycle was chosen (sinus rhythm beat with optimum myocardium throughout diastole and systole). The reference point was placed at the beginning of the QRS. The endocardial border was traced in end-systole, and the region of interest was adjusted to exclude the pericardium by aligning the epicardial border. The integrity of the tracking was visually confirmed as well as ascertained from the credibility of the strain curves, in addition to the automated tracking detection displayed by the software. If necessary, the region of interest was readjusted. Segments with persistently inadequate tracking were excluded from analysis.^16^ Global longitudinal strain (GLS) was calculated from an average of the regional longitudinal strain values in the three apical views. Time to aortic valve opening (AVO) and aortic valve closure (AVC) was measured by pulsed wave Doppler in the left ventricular outflow tract using a 2 mm-sample volume (*Figure 1*).

### Definition of septal deformation patterns

**Figure 2:**
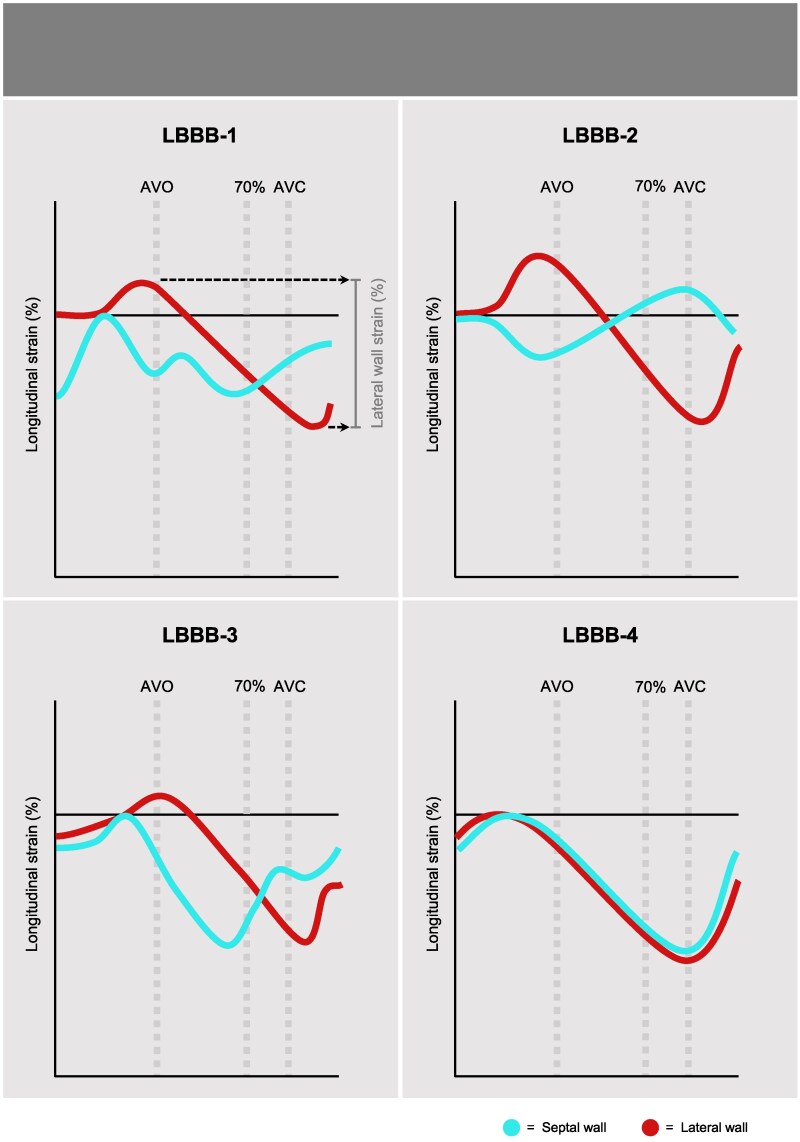
Septal deformation patterns in left bundle branch block. ***LBBB-1:*** Biphasic septal pattern with peaks within 150% of each other. Lateral strain values were measured from the curve zenith to nadir as illustrated. ***LBBB-2:*** Shortening with early peak during pre-ejection phase. ***LBBB-3:*** Shortening with one systolic peak *inside* 70% of ejection phase. ***LBBB-4:*** Shortening with septal peak *outside* the first 70% of ejection phase. AVO; aortic valve opening. AVC; aortic valve closure. The dotted line marked 70% indicates the time to 70% of the ejection phase.

Categorization of septal deformation patterns was predefined according to previously described patterns (*Figure 2*).^11,12^ Longitudinal strain curves of the basal or mid-septal segments in the apical four-chamber view were used to classify the curves into four septal deformation patterns (original clinical tracings are provided in Supplementary data online, *Figures S1–S4*):

**LBBB-1:** Biphasic (double-peaked) was determined if the amplitudes of the peaks were within 150% relative range of each other; in all other cases, the dominant peak was considered the only systolic peak.
**LBBB-2:** Shortening with an early septal peak during the pre-ejection phase, followed by stretch during the rest of the ejection phase.

Based on previous clinical observations, some patients have early septal deformation without meeting the criteria for the LBBB-1 or LBBB-2 pattern. Therefore, we introduce a new pattern, LBBB-3, with one systolic peak occurring relatively early in the ejection phase. The 70% cutoff is based on previous publications where it was observed that patients with a typical LBBB contraction pattern often exhibit septal peak contraction within the first 70% of the ejection phase and that this typical pattern is associated with strict ECG criteria for LBBB.^7^

**LBBB-3:** Shortening with one systolic peak *inside* 70% of the ejection phase (and outside the pre-ejection phase to distinguish from LBBB-2).
**LBBB-4:** Normal timing of the septal peak. There was shortening with the septal peak *outside* the first 70% of the ejection phase.

Deformation patterns have previously been described as typical or atypical based on septal and lateral strain curves.^10^ As lateral wall function modifies the shape of the septal strain curves, classification can be performed by relying solely on septal strain curves. In the context of the previous classification, pattern types LBBB-1, LBBB-2, and LBBB-3 are represented within the spectrum of the typical deformation pattern, whereas LBBB-4 corresponds to an atypical deformation pattern.

### Lateral wall

Lateral wall strain was assessed by measuring the average strain-value of the lateral wall midventricular and basal segments. Values were measured from the curve zenith to nadir as shown in *Figure 2*, LBBB-1.

### Intra- and inter-observer reproducibility

Twenty-five randomly selected studies were evaluated and re-evaluated by the original observer (NR) and independently by a second observer (BT). The intra- and inter-observer concordance on identifying the types of patterns was 25/25 and 24/25, respectively.

### Long-term outcome and subgroup analyses

The primary outcome was a composite of all-cause mortality, heart transplantation, or implantation of a left ventricular assist device (LVAD). Vital status was ascertained through the United States Social Security Death Index and the Danish civil registration register, respectively.

### Statistical analysis

Relevant variables were tested for normality using visual inspection of histogram plots and are presented as mean ± SD. Continuous variables were compared using Student’s *t*-test. Proportional differences were tested using χ^2^ statistics or Fisher’s exact test, where appropriate. Proportional hazards assumptions were verified graphically. For all survival analyses, follow-up was truncated at a maximum of four years (1460 days). The cumulative probability of the endpoint was illustrated using the Kaplan-Meier method with significance testing using log-rank statistics. Univariable and multivariable predictors of event-free survival after CRT-D implantation were assessed in Cox proportional hazards models.

Candidate variables with *P*-values of <0.05 in univariable analysis were included in the multivariable model using backward selection to test the independent association between outcome and lateral wall amplitude as well as type of septal deformation pattern. Due to the interaction between septal pattern and lateral wall performance, these parameters were tested in separate models. A two-tailed *P*-value of <0.05 was considered significant in the final models.

The ability to reclassify patient risk when assessments of patterns were added to the multivariable model was evaluated by assessment of the net reclassification index (NRI) and the integrated diagnostic improvement. Furthermore, the lateral wall performance was tested in a model including septal patterns. Patients were initially classified at a low or high risk of an event if their predicted risk was < or ≥10%, respectively, a predefined cutoff derived from previous studies.^17^

All statistical analyses were performed using SAS for Windows version 9.1.3 (SAS Institute, Cary, North Carolina, USA).

## Results

### Baseline characteristics

Out of 234 patients with native LBBB, 26 patients (11%) were excluded either because of atrial fibrillation (*N* = 7, 3%) or due to poor image quality (*N* = 19, 8%). Accordingly, 208 patients were included; all patients had complete baseline data.

Baseline characteristics according to the type of septal deformation pattern are presented in *Table 1*. Baseline characteristics showed variation according to the type of pattern. Patients with type LBBB-1 or LBBB-2 had an overall wider QRS duration and more often QRS >150 ms in 71% vs. 54% of patients *(P* = *0.02).* More patients were female, 41% vs. 17% *(P*  *<*  *0.001),* and more patients had non-ischaemic cardiomyopathy 57% vs. 31% *(P*  *<*  *0.001),* when compared to patients with type LBBB-3 or LBBB-4.

**Table 1:** Baseline characteristics according to the type of septal contraction pattern

N = 208	LBBB-1 (*n* = 36) Mean ± SD	LBBB-2 (*n* = 51) Mean ± SD	LBBB-3 (*n* = 43) Mean ± SD	LBBB-4 (*n* = 78) Mean ± SD	*P*-value
Age	66 ± 9	65 ± 12	68 ± 9	66 ± 10	0.55
Female, no. (%)	12 (33)	24 (47)	10 (23)	11 (14)	<0.001
NYHA Class	2.7 ± 0,4	2.6 ± 0,4	2.7 ± 0,4	2.7 ± 0,4	0.83
QRS, ms	161 ± 24	164 ± 18	162 ± 27	153 ± 22	0.03
QRS > 150 ms, no.	22 (61)	40 (78)	28 (65)	38 (49)	0.01
Ischaemic etiology	14 (39)	23 (45)	24 (56)	59 (76)	< 0.001
eGFR, mL/min	74 ± 21	72 ± 23	71 ± 24	70 ± 22	0.82
LVEF, %	25 ± 6	21 ± 5	26 ± 6	25 ± 6	0.001
LVESV, mL	144 ± 50	169 ± 73	152 ± 60	154 ± 72	0.38
LVEDV, mL	188 ± 57	213 ± 84	203 ± 73	202 ± 83	0.65
GLS	−9.3 ± 3	−7.6 ± 3	−8.9 ± 3	−9.1 ± 4	0.06
Lateral wall strain	−12.1 ± 4	−10 ± 4	−9.8 ± 4	−9.4 ± 5	0.03
Betablocker, no. %	33 (92)	46 (90)	39 (91)	72 (92)	0.97
ACEi/ARB, no. %	34 (94)	45 (88)	42 (98)	71 (91)	0.34

ACEi/ARB, Angiotensin-converting-enzyme inhibitor/angiotensin receptor blockers; eGFR, Estimated glomerular filtration rate; LVEF, Left Ventricular Ejection Fraction; LVEDV, Left Ventricular End-diastolic Volume; LVESV, Left Ventricular End-systolic Volume; NYHA class, New York Heart Association functional classification for heart failure.

The median follow-up was 4.0 years (interquartile range: 3.25–4 years). During follow-up, 38 patients (18%) died, 4 (2%) had a heart transplant, and 6 (3%) received LVADs. Eleven patients (5%) died during the first 6 months after device implantation.

### Outcome in relation to septal strain patterns

Out of the 208 patients, 36 (17%) had LBBB-1, 51 (25%) had LBBB-2, and 43 (21%) had LBBB-3. Seventy-eight patients (38%) did not have early septal contraction, LBBB-4.

**Figure 3:**
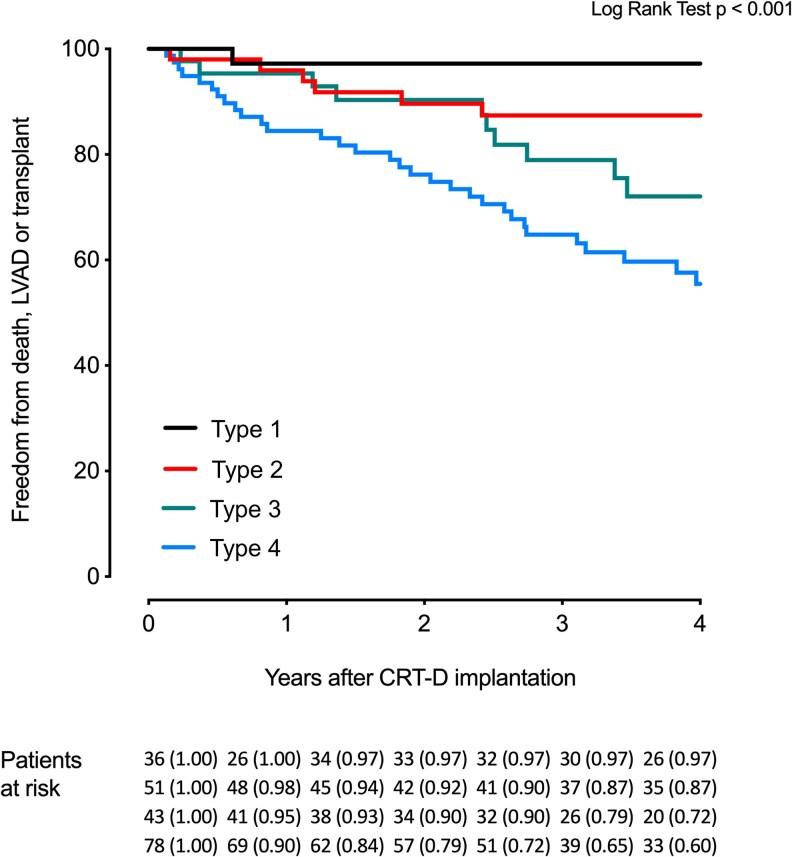
Septal deformation patterns and survival. Kaplan-Meier plot demonstrating survival for different types of septal contractions.

The type of septal deformation pattern at baseline was highly associated with long-term outcome after CRT. Patients with LBBB-4 showed the least favourable outcome and were therefore used as the reference group for further analysis. *Figure 3* shows Kaplan-Meier curves according to septal deformation patterns.

In a univariable model, patients with LBBB-1 and LBBB-2 had one-sixth the risk of the primary outcome compared to those with LBBB-4 (HR: 0.17 [95% CI: 0.07–0.38], *P* < 0.001). A trend towards a lower risk of the primary outcome was found comparing patients with LBBB-3 to patients with LBBB-4 (HR: 0.54 [0.26–1.09], *P* = 0.086).

In multivariable analysis including GLS, LVEF, QRS duration and heart failure aetiology, the presence of a LBBB-1 or LBBB-2 septal deformation pattern remained highly associated with a favourable outcome (HR: 0.14 [0.06–0.34], *P* < 0.001 compared to those with LBBB-4) while a trend towards lower risk remained comparing LBBB-3 to LBBB-4 (HR: 0.62 [0.30–1.30], *P* = 0.21).

### Lateral wall deformation

**Figure 4:**
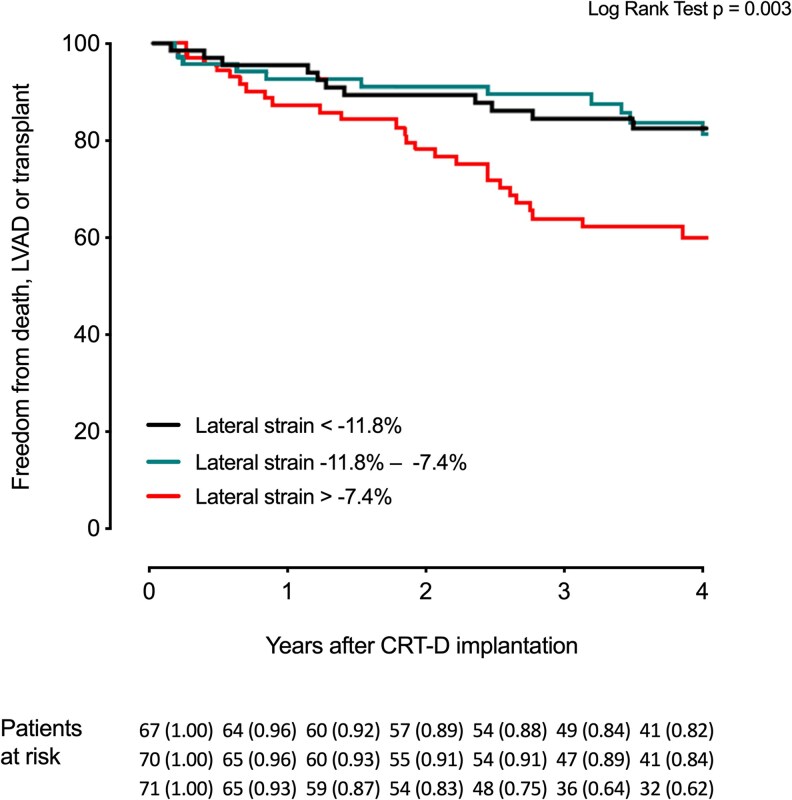
Lateral strain amplitude and survival. Kaplan-Meier plot demonstrating survival for lateral strain amplitude divided in tertiles.

Lateral wall deformation amplitude was found to be significantly associated with outcome. The better the lateral wall deformation, the less the likelihood of an event. On Cox regression, a (numerical) reduction in negative strain (less negative) of 1% was significantly associated with the outcome (HR 1.14 [95% CI 1.06–1.22], *P* < 0.001), and lateral wall strain remained independently associated with outcome in the multivariable model including GLS, LVEF, QRS duration, and heart failure aetiology (HR: 1.13 [95% CI 1.02–1.25], *P* = 0.02). *Figure 4* shows the Kaplan-Meier curves according to tertiles of lateral wall strain.

Patients in the lowest tertile (poor strain > −7.4%) were highly prone to experience an event in comparison to the other patients (38% vs. 17%), HR 2.57 [95% CI 1.46–4.55] (*P* = 0.001).

### Global longitudinal strain

More negative GLS values were associated with event-free survival. A reduction of 1% in negative strain (less negative) was significantly associated with outcome HR 1.11 [95% CI 1.01–1.22] *(P* = *0.03)* with a clear trend in the multivariable model including QRS duration and aetiology (HR 1.09 [95% CI 0.99–1.20] *P* = *0.08*).

When divided into tertiles, a significant difference in outcome was demonstrated between the upper and lower GLS tertiles, HR 2.53 [95% CI 1.17–5.46] *(P* = *0.02).*

**Figure 5:**
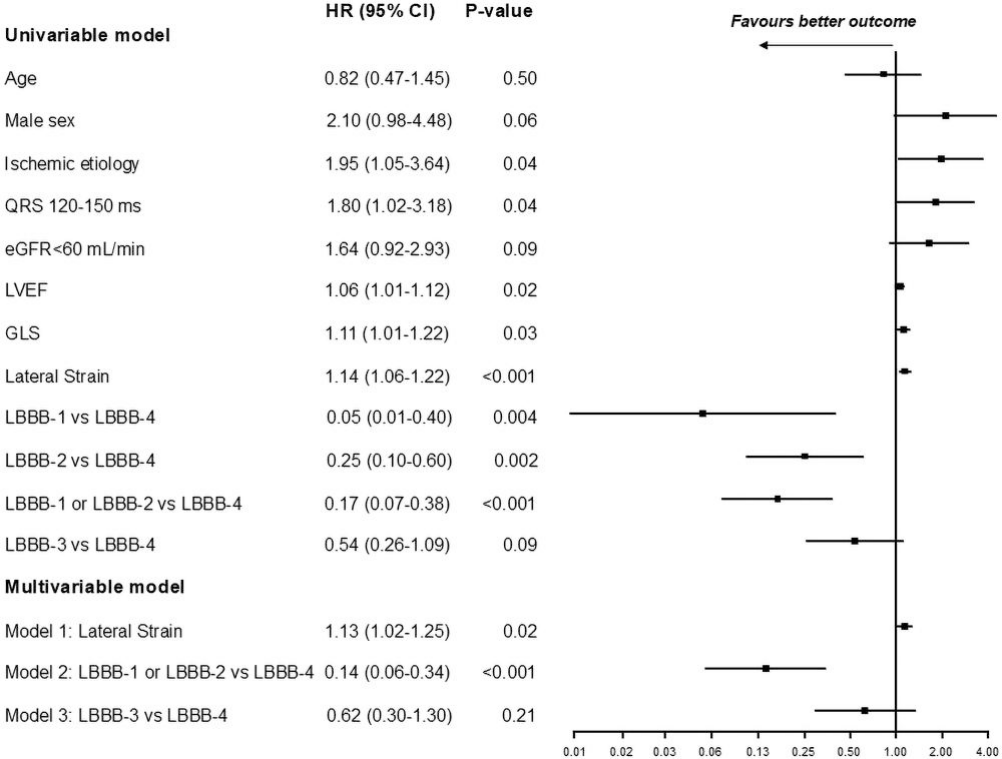
Univariable and multivariable risk analyses of the primary outcome after cardiac resynchronization therapy. Cox proportional hazards modelling with composite outcome of death, Left Ventricular Assist Device or heart transplant. LVEF; Left Ventricular Ejection Fraction, LVEF refers to a reduction of 1% GLS; global longitudinal strain. GLS and lateral strain refers to a reduction in negative strain of 1% (less negative), eGFR; Estimated glomerular filtration rate, LBBB; Left bundle branch block. The multivariable model included: GLS, LVEF, QRS duration and heart failure aetiology.

*Figure 5* shows a Forrest plot of univariable and multivariable risk analyses of the primary outcome after cardiac resynchronization therapy.

### Risk reclassification by adding strain parameters

Adding assessment of GLS to a model including aetiology and QRS duration did not significantly improve risk prediction. C-statistics increased from 0.63 (0.54–0.72) to 0.67 (0.58–0.76) *(P* = *0.13).* However, when adding lateral wall assessment, a significant increase was demonstrated to 0.70 (0.61–0.79) *(P* = *0.046).*

In a model including GLS, aetiology, and QRS duration, C-statistics increased to 0.75 (0.67–0.83) *(P* = *0.02),* when adding septal pattern assessment. There was no added value in lateral wall assessment if the septal pattern configuration was already known *(P* = *0.62).*

NRI analysis showed that adding septal pattern assessment to a 4-year 10% risk model with QRS duration, aetiology, and GLS yielded a significant integrated diagnostic improvement (0.064*; P* = *0.003*) and NRI (0.18; *P* = *0.001*), driven by a correct downward risk classification of 33 patients (4 incorrectly reclassified upward) among those without events *(P*  *<*  *0.001).*

## Discussion

The current study demonstrated that specific features within the LBBB contraction are associated with a favourable response to CRT with 4 years of follow-up. Importantly, this study represents the longest follow-up to date on the association between four different septal deformation patterns and mortality in a CRT-cohort. The type of septal deformation, as well as the lateral wall performance, are important determinants of response and are independently associated with long-term outcome. However, the added value of lateral wall assessment is limited when the septal deformation pattern is known. Furthermore, parameters of regional deformation were found to be superior to global longitudinal deformation in the prediction of response. The current study suggests that a more elaborate analysis of particularly septal wall deformation can be useful for the prediction of outcome and to establish prognostic expectations.

It is well-known from landmark clinical trials that patients with severe symptomatic heart failure and LBBB show the most favourable response to CRT.^2^ However, even within this group, a considerable number of patients appear to show little or no response. One reason is that the 12-lead ECG may be misinterpreted as LBBB in cases where electrocardiographic changes are caused by slowed intraventricular conduction delay, hypertrophy, dilatation, or fascicular block.^18,19^ Hence, electrophysiologic mapping studies have demonstrated that up to a third of patients with LBBB morphology by ECG do not have a significant activation delay.^4,5^ Recent studies demonstrate that methods for identifying LBBB by ECG are highly dependent on the definitions used and that methods do not correlate well.^6^

In an attempt to improve the selection of CRT patients, identification of mechanical dyssynchrony by echocardiography has been suggested.^20,21^ The time-to-peak dyssynchrony methods never found a place in the guidelines because the methods were found too unspecific to distinguish between mechanical dyssynchrony induced by an activation delay and dyssynchrony from other causes.^22^ Indeed, significant time-to-peak differences can be present in the absence of a significant LV activation delay due to scar tissue, loading conditions, or heterogeneities in the failing heart.^22^ In these cases, the substrate for CRT is not clear.

In recent years, echocardiographic methods aimed at identifying the presence of an LV activation delay have been introduced. Growing evidence supports a ‘physiology-based’ approach by which specific motion patterns unique to LBBB activation are used to identify appropriate candidates for CRT.^10,23–25^

### Septal deformation

An LBBB contraction sequence is characterized by a complex interplay of opposing wall motion, including early septal deformation (sometimes followed by pronounced stretching), early free wall stretching, and late peak shortening.^7,8,25^ Computer simulations have demonstrated that within this pattern of opposing wall motion, the exact configuration is mainly determined by regional contractility and the degree of activation delay between walls.^11^ Accordingly, for early septal deformation to occur, whether in the shape of peak shortening during the pre-ejection phase or a biphasic configuration, a significant LV activation delay is mandatory, and regional contractility must be relatively preserved.

In the current study, we found these septal patterns of early septal contraction (LBBB-1 and LBBB-2) to be highly predictive of a favourable outcome, and this is in line with the common understanding that an activation delay and a relatively preserved lateral wall function are required for CRT to be beneficial. Patients with septal deformation pattern type LBBB-1 and LBBB-2, were more likely to be female and have nonischaemic cardiomyopathy. This is in line with the finding that more men have ischaemic heart disease and are therefore prone to have reduced myocardial viability, which would diminish a potential early septal contraction, rendering the septal deformation pattern to type LBBB-3 or LBBB-4. This emphasizes the integrated information on electromechanical function provided by the septal deformation patterns, highlighting the primary advantage of their usage.

The terminology regarding the septal deformation patterns is currently rather confusing, as different classifications have been proposed, and there is no consensus on which patterns to use. Our findings are based on computer simulations and are supported by previous studies indicating that early terminated septal shortening is highly associated with outcome.^13,24^ Similar to the present study, Menet *et al.*^13^ used 2D strain echocardiography to identify patterns LBBB-1 and LBBB-2 and showed a strong association with the occurrence of death or heart failure hospitalization during a median follow-up of 2 years. This study extends these findings in a ‘LBBB-only’ cohort with 4 years of follow-up, with further elaboration of deformation patterns. Calle *et al.*^14,26^ introduced a strain-based classification of the degree of LBBB-induced LV dysfunction and suggested that septal deformation patterns provide insight into the extent to which LBBB contributes to the LV dysfunction. In line with our results, they show that septal strain patterns with early septal deformation (similar to our LBBB-1 and LBBB-2 patterns) indicate a more prominent role of LBBB activation in the development of LV dysfunction and consequently a favourable response to CRT. Thus, despite the confusing terminology, there is general agreement that early septal contraction is associated with a favourable outcome to CRT, whereas late septal peak contraction is associated with poorer outcomes to CRT.

Patterns not meeting criteria for LBBB-1 or -2 have sometimes been referred to as ‘pseudonormal’ or LBBB-3.^12^ However, based on our observations, a considerable number of so-called LBBB-3 patterns show early terminated septal shortening within 70% of the ejection phase, followed by only a minor degree of systolic stretching and without meeting criteria for LBBB-1 and -2. We propose that these patients should be deemed subtype LBBB-3 to distinguish them from type LBBB-4, for which septal shortening is no longer considered early. Indeed, the identification of the LBBB-3 pattern is of clinical interest, as patients with this pattern had a 50% reduced risk of an event during 4 years of follow-up compared to those with LBBB-4. An LBBB-3 pattern may be present with an underlying significant LV activation delay in combination with different degrees of reduced lateral wall contractility as elegantly demonstrated in the work from Leenders *et al.*^11^ We find this to be a prevalent pattern in patients with ischaemic cardiomyopathy.

### Lateral wall performance

We further investigated the importance of lateral wall function for response to CRT. A reduced lateral wall amplitude, in this study, the lower tertile (>−7.4%), was associated with a 2.5-fold increased risk of a poor outcome.

The lateral wall is most often the preferred site for LV-lead placement, and it has been demonstrated that positioning the LV-lead in scar tissue may lead to increased mortality or hospitalization.^27–29^ Patients with scar tissue are less likely to experience reverse remodelling,^29^ and localized areas of scar tissue can interfere with the myocardial activation, resulting in an inefficient activation sequence or may constitute a substrate for arrhythmias. A lateral wall with significant scarring, causing suboptimal thresholds, may force the implanting physician to look to other, less-optimal LV positions. LV lateral wall dysfunction may arise at a time when dilatation of the LV leads to an unbearable LV lateral wall workload. Hence, in some cases, poor lateral wall function may indicate advanced heart failure.^30^

There is an interaction between the septal and lateral wall movement.^31^ A relatively preserved lateral wall function is required for early septal shortening to occur. On the other hand, lateral wall amplitude is likely affected by the degree of prestretching caused by septal contraction. Thus, an increased regional pre-load will (to a certain limit) result in a more forceful and vigorous contraction.^31^ These considerations are reflected in the multivariable Cox regression models that were performed separately for the two markers. From a clinical viewpoint, although lateral wall function was found to be independently associated with outcome, lateral wall assessment does not add significant prognostic information in a model including information on septal pattern configuration. In addition, the proposed −7.4% cutoff should be considered hypothesis-generating and should be validated in other cohorts.

### Global compared to regional strain assessment

Several studies have demonstrated the prognostic importance of GLS for CRT outcome, and GLS has been demonstrated to have superior prognostic value over LVEF.^32,33^ The current study confirms that GLS is a predictor of death, LVAD, or heart transplantation, and low GLS, in particular, may lead to a poor outcome. There may even be a possible negative impact of CRT affecting the ‘sickest’ patients with presumably more extensive structural myocardial damage.^32^

However, the current study further demonstrates that regional strain assessment, whether using information regarding lateral wall function or septal deformation patterns, is superior to GLS for risk prediction before CRT-D implantation. GLS is often reported to reflect the extent of scar tissue and replacement fibrosis in heart failure patients.^30,32^ In the presence of LBBB, however, the relationship may not show a clear inverse correlation as strain values are also dependent on the timing of the regional activation-contraction sequence.

### Perspective

Patients with symptomatic, severe heart failure and LBBB have a class Ia indication for CRT. While this is unlikely to be changed, prognostic expectations can sometimes be challenging by current standards. The current data indicate that the use of strain pattern assessment appears to be a valuable tool beyond ECG morphology and duration and may allow the clinician to present more elaborate patient outcome predictions. It is a simple approach and can be limited to septal pattern assessment. Ideally, this approach may be part of an automated algorithm for pre-CRT risk-scoring and work is currently conducted to investigate the value of this approach. Furthermore, septal pattern recognition may be less affected by inter-vendor variability than absolute strain values, as it relies on temporal and morphological characteristics. However, inter-vendor variability of septal deformation patterns was not investigated in the present study but should be examined in future studies.

### Limitations

First, the present study was not randomized and a comparison to patients without CRT could not be conducted. Accordingly, the treatment effect from CRT could not be ascertained nor could any adverse effects from CRT be established. Second, we did not perform assessment of scar tissue. It may be valuable to combine information on scar and deformation patterns. Third, this study does not provide data on LV reverse remodelling, which should be addressed in future studies. However, previous studies have demonstrated that early septal contraction predicts favourable LV remodelling.^3,12^ Fourth, the types of septal patterns included in the current study were derived from previous publications in this field. There may be other patterns with prognostic significance not addressed here. Fifth, the current analysis can only be applied to the equipment used and may not apply to software packages from other manufacturers. Sixth, as patients with atrial fibrillation were excluded from the analysis, the findings are not applicable to patients with atrial fibrillation. However, assessment of septal deformation patterns may not differ significantly between patients in sinus rhythm. This should, however, be investigated in future studies. Finally, this is a semiquantitative method, and a simple number is difficult to obtain along a continuum of disease. However, as previously stated, we find it highly reproducible.

## Conclusions

The clinical benefit with regard to long-term outcomes in CRT-D patients who have an LBBB prior to CRT implantation varies. Subtypes of septal deformation patterns and, to a lesser degree, lateral function, are important determinants of outcome in patients undergoing CRT-D implantation. The current data indicate that pattern identification by echocardiographic strain analysis may serve as a prognostic separator of patients with LBBB.

## Supplementary Material

qyag066_Supplementary_Data

## Data Availability

Data for this study is not publicly available due to patient privacy concerns.

## Lead author biography

Dr. Risum is a consultant electrophysiologist at Copenhagen University Hospital—Rigshospitalet. His research has primarily focused on cardiac implantable electronic devices, including implantable cardioverter-defibrillators and cardiac resynchronization therapy. A key area of interest has been the interaction between electrical cardiac disease and cardiac mechanical deformation, with two-dimensional strain echocardiography serving as one of the principal research tools. As part of his PhD thesis, he described a strain-based classification of a typical contraction pattern in patients with left bundle branch block undergoing cardiac resynchronization therapy.
