# Supplementary material for: Prognostic importance of septal deformation patterns and lateral wall function in patients with heart failure and left bundle branch block receiving cardiac resynchronization therapy
Source: Eur Heart J Imaging Methods Pract. 2026 May 13;4(1):qyag066. doi: 10.1093/ehjimp/qyag066 (PMC13173436; doi:10.1093/ehjimp/qyag066)
Supplement: qyag066_Supplementary_Data [file qyag066_supplementary_data.docx]

Supplementary material

**Supplementary Figure 1** Original strain curves in a patient with LBBB-1

**
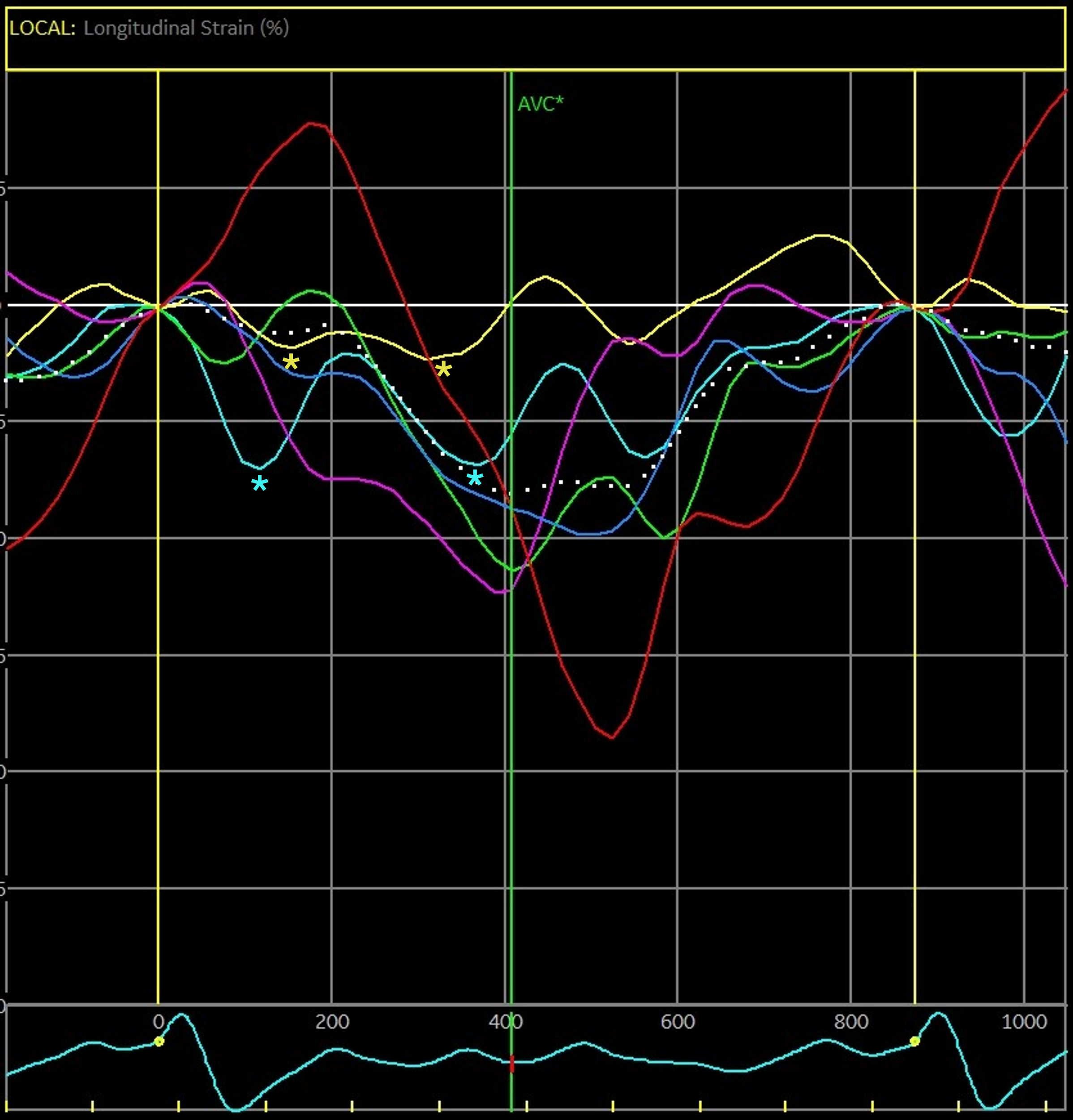
**

Legend for Supplementary Figure 1: Longitudinal strain curves obtained from the apical four-chamber view in a patient with type LBBB-1. The strain curves of the basal (yellow) and mid-ventricular (cyan) septal wall have two systolic peaks (marked with *) within 150% relative range of each other. Therefore, the strain curves are classified as biphasic, LBBB-1.

Yellow curve = basal septal wall, cyan curve = mid septal wall, green curve = apical septal wall, pink curve = apical lateral wall, blue curve = mid lateral wall, red curve = basal lateral wall, dotted line = global strain curve. AVC; aortic valve closure.

**Supplementary Figure 2** Original strain curves in a patient with LBBB-2

**
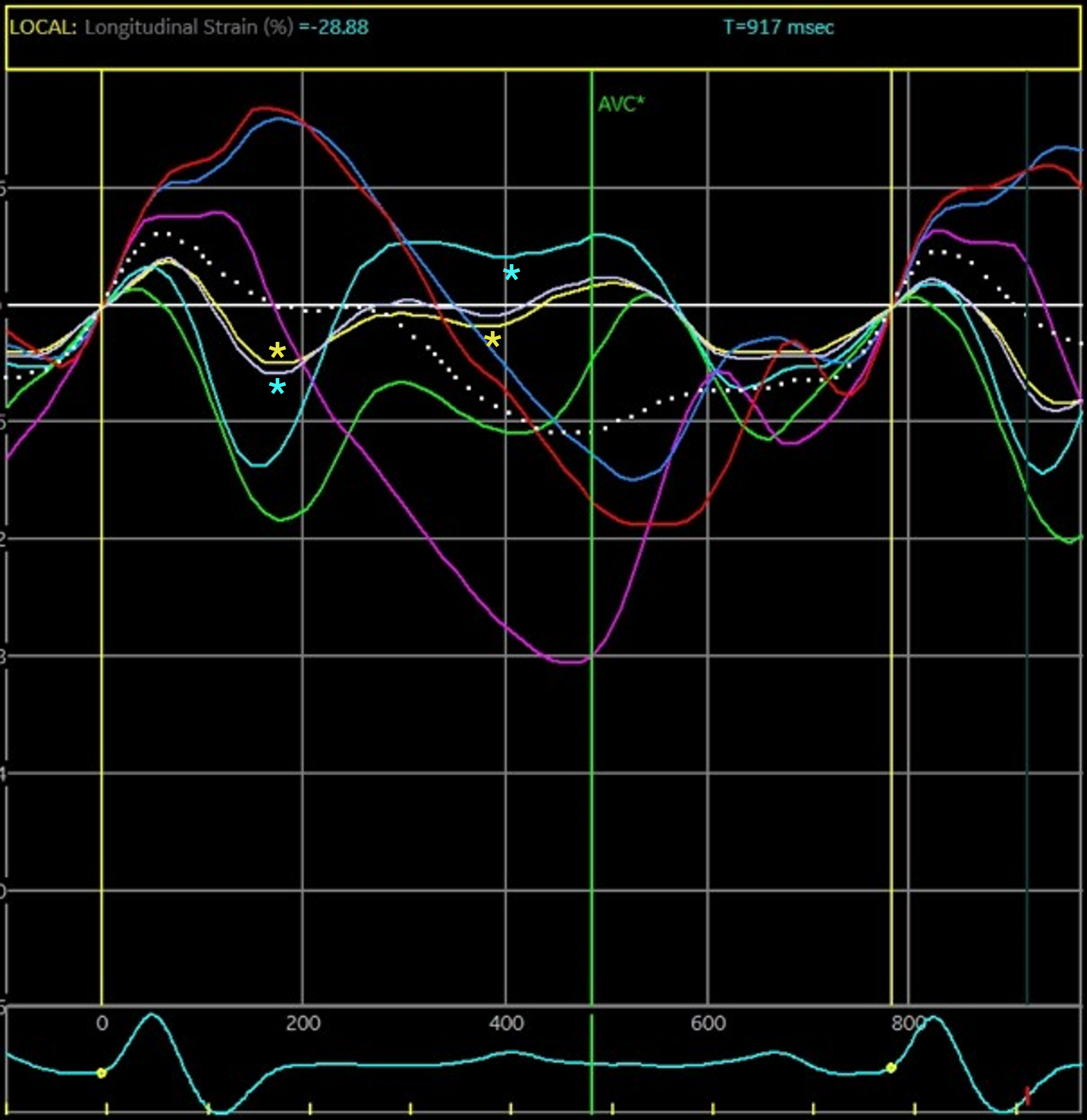
**

Legend for Supplementary Figure 2: Longitudinal strain curves obtained from the apical four-chamber view in a patient with type LBBB-2. Peaks (marked with *) of the basal (yellow) and mid-ventricular (cyan) strain curves are outside 150% relative range of each other so only the first systolic peak is considered. Type LBBB-2 is then identified as the curves peak during pre-ejection followed by stretch during the rest of the ejection phase.

Yellow curve = basal septal wall, cyan curve = mid septal wall, green curve = apical septal wall, pink curve = apical lateral wall, blue curve = mid lateral wall, red curve = basal lateral wall, dotted line = global strain curve. AVC; aortic valve closure.

**Supplementary Figure 3** Original strain curves in a patient with LBBB-3

**
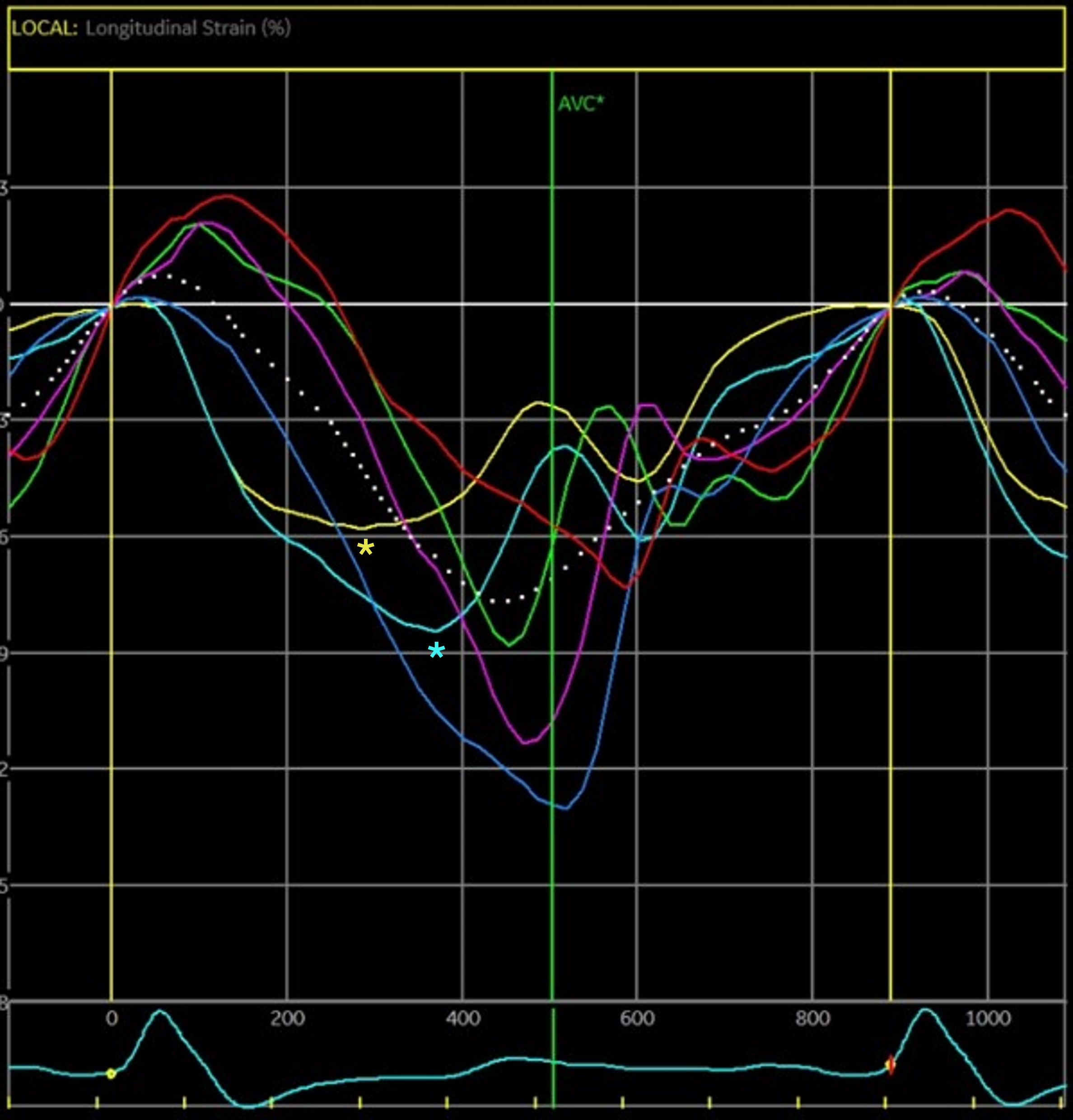
**

Legend for Supplementary Figure 3: Longitudinal strain curves obtained from the apical four-chamber view in a patient with type LBBB-3 as the basal (yellow) and mid-ventricular (cyan) septal strain curves show one systolic peak (marked with *) inside 70% of ejection phase.

Yellow curve = basal septal wall, cyan curve = mid septal wall, green curve = apical septal wall, pink curve = apical lateral wall, blue curve = mid lateral wall, red curve = basal lateral wall, dotted line = global strain curve. AVC; aortic valve closure.

**Supplementary Figure 4** Original strain curves in a patient with LBBB-4

**
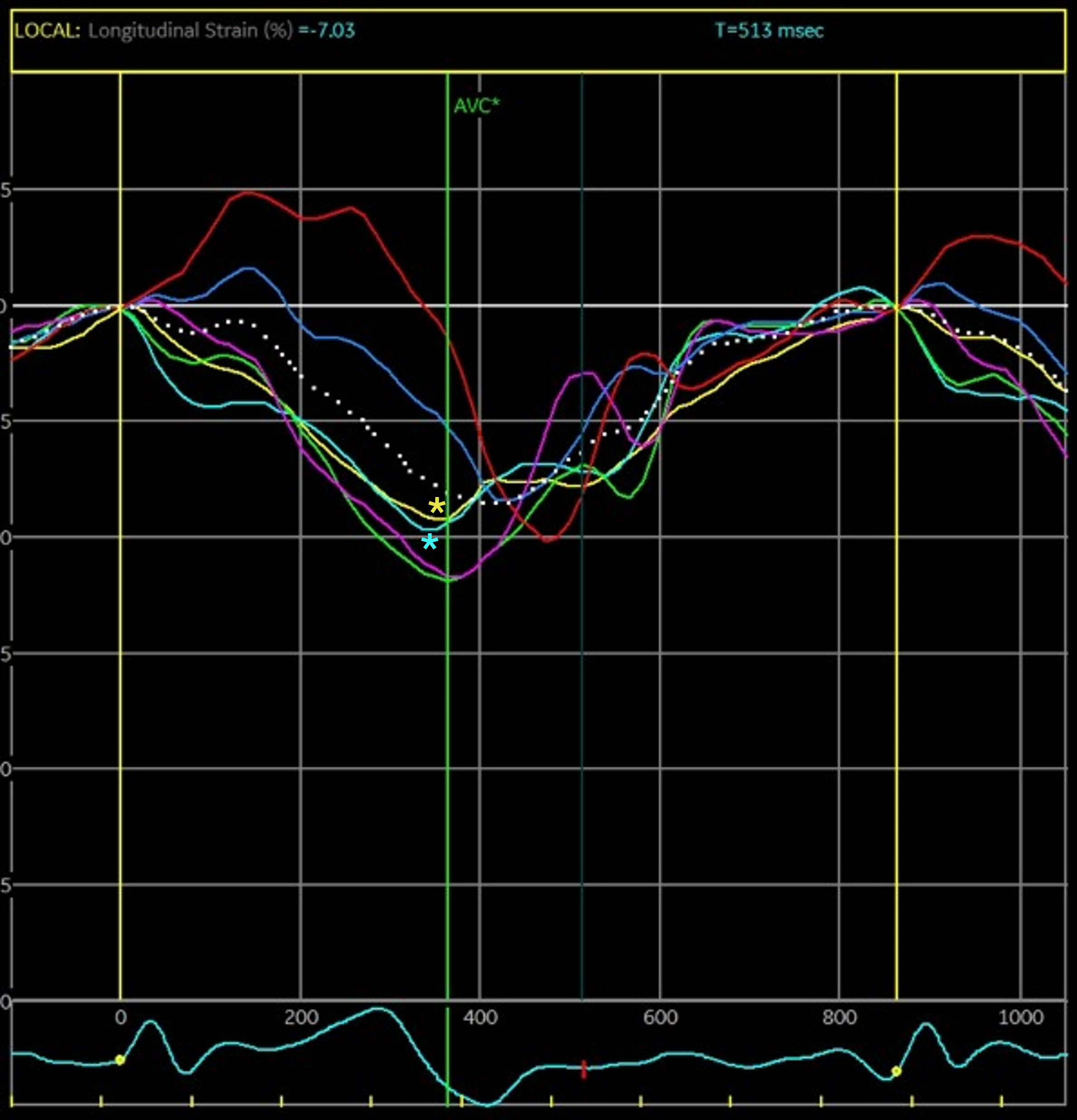
**

Legend for Supplementary Figure 4: Longitudinal strain curves obtained from the apical four-chamber view in a patient with type LBBB-4 as the basal (yellow) and mid-ventricular (cyan) septal strain curves show one systolic peak (marked with *) outside 70% of ejection phase.

Yellow curve = basal septal wall, cyan curve = mid septal wall, green curve = apical septal wall, pink curve = apical lateral wall, blue curve = mid lateral wall, red curve = basal lateral wall, dotted line = global strain curve. AVC; aortic valve closure.
